# Relationship of Hemoglobin A1c with *β* Cell Function and Insulin Resistance in Newly Diagnosed and Drug Naive Type 2 Diabetes Patients

**DOI:** 10.1155/2016/8797316

**Published:** 2015-11-10

**Authors:** Xinguo Hou, Jinbo Liu, Jun Song, Chuan Wang, Kai Liang, Yu Sun, Zeqiang Ma, Weifang Yang, Chengqiao Li, Xiuping Zhang, Peng Lin, Lei Gong, Meijian Wang, Fuqiang Liu, Wenjuan Li, Fei Yan, Jun Qin, Lingshu Wang, Jidong Liu, Ruxing Zhao, Shihong Chen, Li Chen

**Affiliations:** ^1^Department of Endocrinology, Qilu Hospital, Shandong University, Jinan, Shandong 250012, China; ^2^China National Heavy Duty Truck Group Corporation Hospital, Jinan, Shandong 250116, China; ^3^Lukang Hospital of Jining, Jining, Shandong 272141, China; ^4^Department of Endocrinology, Second People's Hospital of Jining, Jining, Shandong 272049, China; ^5^Shantui Community Health Center, Jining, Shandong 272000, China; ^6^Department of Endocrinology, The Second Hospital of Shandong University, Jinan, Shandong 250012, China

## Abstract

*Objective*. To investigate changes in the glycated hemoglobin A1c (A1c) level and those in *β* cell function and insulin resistance in newly diagnosed and drug naive type 2 diabetes patients and to evaluate the relationship between them. *Design and Methods*. A total of 818 newly diagnosed diabetic individuals who were ≥40 years of age were recruited. The subjects were grouped by A1c values (<6.5%, 6.5–7%, 7-8%, 8-9%, and ≥9%). The homeostasis model assessment (HOMA) was used to evaluate pancreatic *β* cell function (HOMA-*β*) and insulin resistance (HOMA-IR). ANOVA, *t*-tests, and binary logistic regression analysis were used for data analysis. *Results*. Compared with subjects with A1c values <6.5%, individuals with an A1c of 6.5–7% exhibited an increased HOMA-*β* index. However, the HOMA-*β* index was significantly decreased at A1c values ≥7% and further decreased by 9.3% and by 23.7%, respectively, at A1c values of 7-8% and 8-9%. As A1c increased to ≥9%, a 62% reduction in *β* cell function was observed, independently of age, gender, body mass index (BMI), blood pressure (BP), blood lipids, and hepatic enzyme levels. Meanwhile, insulin resistance was significantly increased with an increase in A1c values. *Conclusions*. Elevated A1c values (≥7%) were associated with substantial reductions in *β* cell function.

## 1. Introduction

The main pathophysiological defects responsible for type 2 diabetes mellitus (T2DM) include *β* cell dysfunction and decreased insulin sensitivity [1]. In the presence of insulin resistance, progressive loss of *β* cell function is a crucial defect [2]. Many factors including hyperglycemia and elevated free fatty acid accelerate *β* cell deterioration [3]. Accumulating evidence has shown that sustained hyperglycemia is deleterious to *β* cell function. The hemoglobin A1c (A1c) value is an integrated measure of mean glycemia over the preceding 8–12 weeks and is considered the “gold standard” for monitoring metabolic control in subjects with diabetes [4]. It has been reported that an increase in the A1c level is usually accompanied by a decline in pancreatic *β* cell function. However, little is known about the relationship between the A1c level and *β* cell function, especially in newly diagnosed and drug naive type 2 diabetic patients. This study was performed to investigate the changes in A1c along with *β* cell function and insulin resistance in newly diagnosed and drug naive type 2 diabetic patients and to evaluate the relationship between them.

## 2. Materials and Methods

### 2.1. Ethics Statement

The present work consists of one part of the baseline survey from the Risk Evaluation of cAncers in Chinese diabeTic Individuals: a lONgitudinal (REACTION) study, which was conducted among 259,657 adults, aged 40 years and older, in 25 communities across mainland China from 2011 to 2012 [5–8]. This study was approved by the Ruijin Hospital Ethics Committee of the Shanghai Jiao Tong University School of Medicine. Written informed consent was obtained from the study participants.

### 2.2. Study Population

A total of 10,028 subjects were recruited (40 years of age and older) in Shandong province from January to April 2012. Based on previous medical histories and OGTT, we selected 818 newly diagnosed and drug naive type 2 diabetes patients. The exclusion criteria consisted of (1) previously diagnosed hepatic disease, including fatty liver, liver cirrhosis, and autoimmune hepatitis; (2) previously diagnosed diabetes; and (3) any malignant disease. A total of 818 subjects (508 women) were eligible for the analysis.

### 2.3. Data Collection

The demographic characteristics, lifestyle, and previous medical histories were obtained by trained investigators through a standard questionnaire. All subjects underwent a baseline evaluation including body mass index (BMI), waist circumference (WC), and blood pressure (BP). Laboratory evaluations of fasting blood glucose (FBG), fasting insulin, cholesterol, triglyceride, ALT, and AST levels were also performed. OGTTs were conducted in all patients, using a glucose load containing the equivalent of 75 g of anhydrous glucose dissolved in water. The A1c level was measured by high-performance liquid chromatography (VARIANT II and D-10 Systems, BIO-RAD, USA). The homeostasis model assessment of insulin resistance (HOMA-IR) index was calculated as follows: fasting insulin concentration (mIU/L) × FBG concentration (mmol/L)/22.5. The HOMA-*β* index was calculated as follows: 20*∗*fasting insulin concentration (mIU/L)/(FBG concentration (mmol/L) − 3.5) [9].

### 2.4. Definition

Diabetic patients who were diagnosed based on the 1999 World Health Organization (WHO) criteria (FBG ≥ 126 mg/dL (7.0 mmol/L) and/or 2hPG ≥ 200 mg/dL (11.1 mmol/L)) [10] were identified after OGTTs. To explore the association between A1c and insulin resistance/*β* cell function, we divided the subjects into the following five groups according to the A1c values: <6.5%, 6.5–7%, 7-8%, 8-9%, and ≥9%.

### 2.5. Statistical Analysis

Continuous variables with a normal distribution are expressed as the means ± standard deviation (SD), and variables with a nonnormal distribution are presented as medians (interquartile range). Categorical variables are presented as numbers (%). Between-group differences were evaluated with ANOVA. Binary logistic regression analysis was used to estimate the association between A1c levels and *β* cell function/insulin resistance in three models. The following three models were constructed: Model 1 = not adjusted; Model 2 = adjusted for age, gender, BMI, and WC; Model 3 = Model 2 plus systolic BP, diastolic BP, cholesterol, triglycerides, ALT, and AST values. A value of *P* < 0.05 was considered statistically significant. The data were analyzed using the SPSS 16.0 software (SPSS, Inc., Chicago, IL, USA).

## 3. Results

### 3.1. Characteristics of Study Participants Grouped by A1c Category

We recruited a total of 818 newly diagnosed and drug naive diabetic subjects, including 508 females and 310 males with an average age of 60.4 ± 9.6 years. The subjects were divided into five groups according to their A1c levels. As shown in Table 1, no difference in gender, systolic BP, cholesterol, LDL-C, and AST levels were observed between groups. Individuals with an A1c of 7-8% were more likely to be older and stronger and have a higher diastolic BP than those with an A1c < 6.5%.

### 3.2. *β* Cell Function and Insulin Resistance Changes in Different A1c Groups

**Figure 1:**
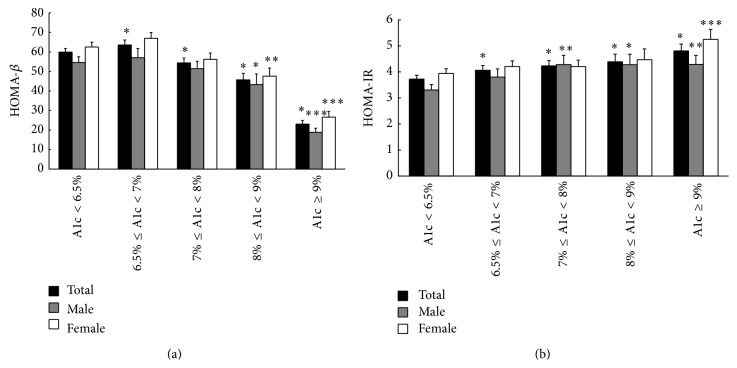
(a) *β* cell function with different A1c groups (HOMA-*β*). (b) Insulin resistance with different A1c groups (HOMA-IR).

We used the HOMA-*β* index to assess *β* cell function. As shown in Figure 1(a), compared to subjects with an A1c < 6.5%, individuals with an A1c of 6.5–7% exhibited increased *β* cell function. By contrast, the HOMA-*β* index was significantly decreased in individuals with an A1c ≥ 7%. As A1c increased to ≥9%, a 62% reduction in *β* cell function was observed. We further compared *β* cell function at different A1c levels in male and female subjects. Impaired *β* cell function was observed in subjects with an A1c ≥ 8% in both male and female patients; the values were decreased by 26% and 48%, respectively. Furthermore, the HOMA-*β* index values in female patients were significantly higher than those in male patients with an A1c ≥ 9%.

We further used the HOMA-IR index to assess insulin resistance in different A1c groups (Figure 1(b)). Insulin resistance increased significantly with increasing A1c levels. Compared with the A1c < 6.5% group, insulin resistance increased by 9%, 14%, 18%, and 29% in individuals with A1c values of 6.5–7%, 7-8%, 8-9%, and ≥9%, respectively. In male patients, insulin resistance was significantly higher in individuals with an A1c ≥ 7% than in individuals with an A1c < 6.5%, while in female patients, the HOMA-IR index was increased only in individuals with an A1c ≥ 9%. No significant difference was observed between male and female patients.

### 3.3. Binary Logistic Regression Analysis

As shown in Table 3, we analyzed the association between increased A1c levels and impaired *β* cell function using three models. We found that patients with A1c values of 8%-9% and ≥9% had a significantly decreased *β* cell function (odds ratio (OR) = 2.45 and 15.36, resp.). After adjusting for age, gender, BMI, and WC, these two groups still presented increased ORs (3.69 and 22.08, resp.). After further adjusting for systolic BP, diastolic BP, cholesterol, triglycerides, ALT, and AST, the patients with A1c levels of 8%-9% and ≥9% also showed an increased risk of impaired *β* cell function (OR = 4.19 and 28.51, resp.).

Similarly, we analyzed the association between increased A1c levels and insulin resistance using the three models (Table 2). As expected, the A1c value was significantly increased with increased insulin resistance. In Model 1, the patients with A1c values of 8%-9% and ≥9% had a significantly increased risk of insulin resistance (odds ratio (OR) = 1.89 and 1.85, resp.). After adjusting for age, gender, BMI, and WC, group 5, with an A1c ≥ 9%, also presented an increased OR (2.16). After further adjusting for systolic BP, diastolic BP, cholesterol, triglycerides, ALT, and AST, patients with an A1c value of 9% also showed an increased risk of insulin resistance (OR = 2.04, *P* = 0.014).

## 4. Discussion

The prevalence of diabetes has increased significantly in recent decades and is now reaching epidemic proportions in China. The most recent national survey in 2013 reported that the prevalence of diabetes and prediabetes in Chinese adults was 11.6% and 50.1%, respectively [11]. The main pathophysiological defects responsible for type 2 diabetes mellitus (T2DM) are *β* cell dysfunction and decreased insulin sensitivity. Pancreatic *β* cell dysfunction plays a major role in determining dysglycemia from the onset of diabetes [12, 13]. Studies from UKPDS have documented a reduction in *β* cell function of up to 50% at the time of diagnosis, and this value gradually increases with the progression of diabetes [14]. Increasing evidence suggests that *β* cell function protection should be a priority starting at the onset of diabetes.

The A1c is an integrated measure of mean glycemia over the preceding 8–12 weeks, and the ADA has recommended the A1c value as a diabetes diagnosis standard [15]. Previous studies have shown that an increase in the A1c level is usually accompanied by a decline in pancreatic *β* cell function. However, whether this trend is present in newly diagnosed and drug naive diabetes patients remains unclear. Moreover, *β* cell function changes at different A1c levels, and these changes in newly diagnosed T2DM patients have not been characterized. In this study, we divided the subjects into five groups based on their A1c levels and compared *β* cell function and insulin resistance at different A1c levels. We found that individuals with an A1c of 6.5–7% exhibited an increased HOMA-*β* index compared with subjects who had an A1c < 6.5%, indicating that a slight increase in the A1c level may induce increases in insulin secretion, which occur to compensate for rising insulin resistance. However, the HOMA-*β* index was significantly decreased in patients with A1c levels ≥ 7%; it was decreased by 9.3% in patients with an A1c of 7-8% and by 23.7% in patients with an A1c 8-9%. As the A1c level increased to ≥9%, a 62% reduction in *β* cell function was observed, which suggests that poor glycemic control may contribute to the decrease in *β* cell function. These results are consistent with a previous study [16]. Because the A1c value was significantly correlated with *β* cell function in newly diagnosed and drug naive type 2 diabetes patients, our present finding could have potentially important clinical implications. Attention should be focused on the A1c value to protect *β* cell function in diabetic patients. The underlying mechanisms of this process may be found in diabetic rodent studies. Sustained hyperglycaemia damages *β* cell function via several mechanisms such as an increase in oxidative stress, activation of the MAPK pathway, and reduction of the pancreatic and duodenal homeobox factor-1 (PDX-1) function [17, 18].

In addition to hyperglycemia, the traditional risk factors of impaired *β* cell function include age, obesity, hypertension, ALT, AST, and dyslipidemia. We adjusted for age, gender, BMI, systolic BP, diastolic BP, ALT, and AST, as well as WC, cholesterol, and triglyceride levels to ensure that our results were more reliable. After adjusting for the above risk factors, the A1c value was still significantly associated with impaired *β* cell function in patients with A1c levels ≥ 7%; no changes were observed in individuals with an A1c < 7%. To date, no study has detailed insulin sensitivity in Chinese subjects with newly presented type 2 diabetes mellitus stratified by A1c levels. Our study also demonstrated that insulin resistance increased with increasing A1c levels, compared to individuals with an A1c < 6.5%.

Our study also has some limitations. First, our study included only middle-aged and elderly Chinese subjects; therefore, the results might not be applicable to subjects of different ages or ethnicities. Second, we used the HOMA-IR index to evaluate insulin resistance instead of the “gold standard” (euglycemic-hyperinsulinemic clamp techniques). The HOMA-IR index is a mathematical model of the fasting state, and it represents hepatic insulin resistance only; therefore, it cannot reflect insulin resistance accurately. Third, this is only a cross-sectional study. Long-term prospective studies are needed to clarify the association between the changes in *β* cell function and A1c levels during the development of diabetes.

## 5. Conclusions

Elevated A1c levels (≥7%) were associated with substantial reductions in *β* cell function. The A1c value could be used as simple and practical index to evaluate *β* cell function and direct clinical treatment in newly diagnosed and drug naive type 2 diabetes patients.

## Figures and Tables

**Table 1:** Characteristic of study participants grouped by A1c category.

Characteristics	Group 1	Group 2	Group 3	Group 4	Group 5	Total
*N*	311	156	172	67	112	818
Female (%)	198 (63.7%)	103 (66.0%)	109 (63.4%)	38 (56.7%)	60 (53.6%)	508 (62.1%)
Age (years)	59.44 ± 9.91	61.7 ± 9.69	61.56 ± 9.23^*^	61.76 ± 8.72	58.55 ± 8.77	60.39 ± 9.54
BMI (kg/m^2^)	26.44 ± 3.51	27.72 ± 3.19^**^	27.99 ± 3.31^***^	27.56 ± 3.38	26.36 ± 2.86	27.09 ± 3.39
Wc (cm)	87.7 ± 10.16	91.53 ± 8.88^***^	91.51 ± 10.25^***^	91.63 ± 10.84^**^	89.56 ± 8.71	89.81 ± 09.95
Systolic BP (mmHg)	146.15 ± 20.63	146.56 ± 19.42	145.76 ± 19.85	146.31 ± 18.83	147.99 ± 21.11	146.4 ± 20.13
Diastolic BP (mmHg)	83.96 ± 12.49	82.4 ± 11.76	81.02 ± 11.34^**^	80.31 ± 10.69^*^	84.22 ± 12.38	82.78 ± 12.02
FBG (mmol/L)	7.47 ± 1.58	7.4 ± 0.91	7.96 ± 1.14^**^	8.86 ± 1.73^***^	12.54 ± 3.38^***^	8.37 ± 2.47
Fasting insulin (uU/mL)	9.7 (7–13.95)	11.3 (7.8–15.8)	9.7 (6.93–14.6)^*^	9.9 (7–14.8)	7.4 (5.23–11.1)^**^	9.85 (6.9–14)
A1c (%)	5.96 ± 0.34	6.7 ± 0.14^***^	7.39 ± 0.3^***^	8.33 ± 0.28^***^	10.86 ± 1.75^***^	7.27 ± 1.75
Cholesterol (mmol/L)	5.51 ± 1.14	5.68 ± 1.01	5.6 ± 0.97	5.58 ± 0.95	5.76 ± 1.42	5.6 ± 1.11
Triglycerides (mmol/L)	1.42 (1.01–2.1)	1.61 (1.19–2.27)	1.73 (1.26–2.47)	1.91 (1.3–2.8)^*^	1.81 (1.28–2.36)^***^	1.59 (1.12–2.27)
LDL-C (mmol/L)	3.3 ± 0.89	3.45 ± 0.84	3.4 ± 0.83	3.32 ± 0.83	3.64 ± 0.95	3.4 ± 0.88
HDL-C (mmol/L)	1.55 ± 0.39	1.44 ± 0.27^**^	1.36 ± 0.28^***^	1.34 ± 0.24^***^	1.48 ± 0.46	1.46 ± 0.36
ALT (U/L)	13.67 ± 9.75	15.58 ± 11.06^**^	16.94 ± 13.29	16.19 ± 9.89	15.71 ± 10.08	15.21 ± 10.94
AST (U/L)	20.74 ± 9.32	21.13 ± 9.03	22.434 ± 9.67	21.64 ± 11.32	19.27 ± 8.97	21.03 ± 9.49

Data are mean ± SD or median (interquartile range) or number (%). BMI, body mass index; WC, waist circumference; BP, blood pressure; FBG, fasting blood glucose; LDL-C, low-density lipoprotein cholesterol; HDL-C, high-density lipoprotein cholesterol; ALT, alanine aminotransferase; AST, aspartate aminotransferase. ^*^ *P* < 0.05 compared with Group 1; ^**^ *P* < 0.01 compared with Group 1; ^***^ *P* < 0.01 compared with Group 1.

**Table 2:** Logistic regression analysis of the association between different A1c groups and insulin resistance.

Characteristics	Model 1		Model 2		Model 3	
	OR (95% CI)	*P *	OR (95% CI)	*P *	OR (95% CI)	*P *
A1c groups						
Group 1	1 (reference)		1 (reference)		1 (reference)	
Group 2	1.30 (0.82–2.05)	0.264	0.95 (0.57–1.57)	0.834	0.91 (0.54–1.53)	0.708
Group 3	1.29 (0.83–2.00)	0.266	1.03 (0.64–1.68)	0.892	1.01 (0.61–1.68)	0.969
Group 4	1.89 (1.06–3.37)	**0.032**	1.66 (0.86–3.21)	0.134	1.55 (0.76–3.17)	0.226
Group 5	1.85 (1.13–3.02)	**0.014**	2.16 (1.26–3.69)	0.005	2.04 (1.16–3.61)	**0.014**
Female	—		1.79 (1.22–2.63)	**0.003**	2.30 (1.50–3.51)	**<0.001**
Age (years)	—		0.98 (0.96–0.99)	**0.010**	1.00 (0.98–1.02)	0.882
BMI (kg/m^2^)	—		1.13 (1.05–1.21)	**0.001**	1.11 (1.03–1.19)	**0.007**
WC (cm)	—		1.05 (1.02–1.07)	**<0.001**	1.05 (1.02–1.07)	**0.001**
Systolic BP (mmHg)	—		—		1.00 (0.98–1.02)	0.772
Diastolic BP (mmHg)	—		—		1.03 (1.01–1.06)	**0.001**
Cholesterol (mmol/L)	—		—		0.97 (0.80–1.19)	0.787
Triglycerides (mmol/L)	—		—		1.17 (1.02–1.33)	**0.021**
AST (U/L)	—		—		1.01 (0.98–1.04)	0.646
ALT (U/L)	—		—		1.02 (0.99–1.05)	0.118

Model 1: unadjusted. Model 2: adjusted for age, gender, BMI, and WC. Model 3: adjusted for age, gender, BMI, WC, Systolic BP, Diastolic BP, Cholesterol, Triglycerides, AST, and ALT.

**Table 3:** Logistic regression analysis of the association between different A1c groups and impaired *β* cell function.

Characteristics	Model 1		Model 2		Model 3	
	OR (95% CI)	*P *	OR (95% CI)	*P *	OR (95% CI)	*P *
A1c groups						
Group 1	1 (reference)		1 (reference)		1 (reference)	
Group 2	0.80 (0.45–1.40)	0.427	1.22 (0.65–2.28)	0.536	1.33 (0.70–2.53)	0.377
Group 3	1.47 (0.92–2.37)	0.111	2.47 (1.42–4.27)	**0.001**	2.79 (1.58–4.94)	**<0.001**
Group 4	2.45 (1.35–4.48)	**0.003**	3.69 (1.82–7.48)	**<0.001**	4.19 (1.99–8.79)	**<0.001**
Group 5	15.36 (9.05–26.07)	**0.014**	22.08 (11.86–41.12)	**<0.001**	28.51 (14.53–55.95)	**<0.001**
Female	—		0.38 (0.25–0.59)	**<0.001**	0.39 (0.25–0.61)	**<0.001**
Age (years)	—		0.99 (0.97–1.01)	0.356	0.99 (0.97–1.02)	0.398
BMI (kg/m^2^)	—		0.76 (0.70–0.83)	**<0.001**	0.77 (0.70–0.84)	**<0.001**
WC (cm)	—		0.99 (0.96–1.02)	0.405	0.99 (0.96–1.02)	0.455
Systolic BP	—		—		0.99 (0.98–1.01)	0.290
Diastolic BP	—		—		1.01 (0.99–1.03)	0.545
Cholesterol	—		—		0.96 (0.77–1.19)	0.699
Triglycerides	—		—		0.86 (0.73–1.02)	0.083
AST	—		—		1.03 (0.99–1.06)	0.155
ALT	—		—		0.98 (0.95–1.01)	0.137

Model 1: unadjusted. Model 2: adjusted for age, gender, BMI, and WC. Model 3: adjusted for age, gender, BMI, WC, Systolic BP, Diastolic BP, Cholesterol, Triglycerides, AST, and ALT.
